# CD68-Negative Histiocytoses with Cardiac Involvement, Associated with COVID-19

**DOI:** 10.3390/ijms251810086

**Published:** 2024-09-19

**Authors:** Lubov Mitrofanova, Lubov Korneva, Igor Makarov, Maria Bortsova, Maria Sitnikova, Daria Ryzhkova, Dmitry Kudlay, Anna Starshinova

**Affiliations:** 1 Almazov National Medical Research Centre, 197341 St. Petersburg, Russia; mitrofanova_lb@almazovcentre.ru (L.M.); korneva_lo@almazovcentre.ru (L.K.); bortsova_ma@almazovcentre.ru (M.B.); sitnikova_myu@almazovcentre.ru (M.S.); d_ryjkova@mail.ru (D.R.); 2 Department of Pharmacognosy and Industrial Pharmacy, Faculty of Fundamental Medicine, Lomonosov Moscow State University, 119991 Moscow, Russia; d624254@gmail.com; 3 Institute of Immunology, 115478 Moscow, Russia; 4 Department of Mathematics and Computer Science, St-Petersburg State University, 199034 St. Petersburg, Russia

**Keywords:** CD68, Erdheim–Chester disease, xanthogranulomatosis, pericarditis, myocarditis, COVID-19

## Abstract

Histiocytoses are rare diseases characterised by infiltration of affected organs by myeloid cells with a monocyte or dendritic cell phenotype. Symptoms can range from self-resolving localised forms to multisystemic lesions requiring specific treatment. To demonstrate extremely rare cases of CD68-negative cardiac histiocytosis with expression of SARS-CoV-2 antigen in infiltrate cells. We demonstrated a case of Erdheim–Chester disease in a 67-year-old man with pericardial involvement and positive dynamics with vemurafenib treatment, an autopsy case of xanthogranulomatous myopericarditis in a 63-year-old man, surgical material of xanthogranulomatous constrictive pericarditis in a 57-year-old man, and an autopsy case of xanthogranulomatosis in a 1-month-old girl. In all cases, xanthogranuloma cells expressed CD163, many of them spike protein SARS-CoV-2, while CD68 expression was detected only in single cells. In this article, we demonstrated four cases of extremely rare CD68-negative cardiac xanthogranulomatosis in three adults and one child with expression of the spike protein SARS-CoV-2 in M2 macrophages. This potential indirect association between COVID-19 and the development of histiocytosis in these patients warrants further investigation. To substantiate this hypothesis, more extensive research is needed.

## 1. Introduction

Histiocytoses are rare diseases characterised by the accumulation of macrophages, dendritic cells or cells of monocytic origin in various tissues and organs. More than 100 different subtypes of these diseases have been described. The revised 2016 WHO classification has five groups: (1) L-group (Langerhans)—diseases related to Langerhans cell histiocytosis (LCH), dendritic cell tumour unspecified, non-Langerhans cell histiocytosis—Erdheim–Chester disease (ECD), mixed variant of LCH/ECD; (2) C-group (Cutaneous)—cutaneous non-Langerhans cellular histiocytoses, familial xanthogranuloma, including juvenile xanthogranuloma; (3) R-group (Rosai–Dorfman)—diseases related to Rosai–Dorfman disease (sporadic, extranodal, classical); (4) M-group (Malignant)—primary/secondary malignant histiocytosis; (5) H-group (Nemophagocytic lymphohistiocytosis)—diseases related to haemophagocytic lymphohistiocytosis, macrophage activation syndrome. This group is a form of primary immunodeficiency and also belongs to the group of immune dysregulations [1,2].

Langerhans cell histiocytosis (LCH), formerly called histiocytosis X, is the most frequent disease of the histiocytosis group and morphologically manifests itself by infiltration of organs by CD207+ cells with granuloma formation. Pathological foci are most often found in bone tissue, skin, lung, and pituitary gland, and involvement of the liver, spleen, and bone marrow is an unfavourable prognostic factor [3]. The disease is more common in children (1–3 years of age) [4].

Langerhans cells are resident antigen-presenting epidermal cells expressing langerin (CD207), a C-type lectin receptor involved in the formation of unique organelles of endosomal origin, Birbeck granules [5]. The aetiology of LCG is not precisely clear and is a subject of debates. According to Allen CE et al., LCH is reactive in nature and is caused by an aberrant interaction between T-lymphocytes and Langerhans cells. This is evidenced by the detection of different immune cells in the infiltrate and the presence on pathogenic cells of molecules involved in the recruitment and activation of T cells [6]. Badalian-Very G et al., however, identify clonal expansion of histiocytes as a decisive etiological factor and therefore refer LCH to the group of myeloid cell neoplasms. This hypothesis is supported by the fact that in 57% of LCC cases, a mutation has been identified in the BRAF gene, which results in the replacement of glutamate with valine in the 600 codon—BRAF V600E [7]. The manifestations of LGC are diverse and often overlap with other diseases, and the clinical presentation at the onset of the disease varies from self-resolving monogenic forms to multisystem manifestations requiring chemotherapy or specific treatment.

Erdheim–Chester disease is a rare disorder often overlooked by physicians, manifesting mostly in middle age (median age 53 years). More than half of patients with BES have a mutation in the BRAF gene in affected tissues and circulating monocytes that causes a substitution of glutamic acid for valine at position 600 of the BRAF V600E protein [8]. Other mutations such as PIK3CA and NRAS, in which the activation of the RAS-RAF-MEK-ERK pathway through RAS-PI3K-AKT signalling plays a central role, are less frequent [9,10,11]. As we know, BSE is extremely rare and the clinical manifestations can have a wide spectrum. It is difficult to diagnose in practice. BSE diagnosis may take months or even years after the first symptoms of the disease before verification. An important criterion in the diagnosis of BEC is the result of histological and immunohistochemical studies, which reveal tissue infiltration by foamy histiocytes, with so-called giant cells of the Tuton type and an accompanying nonspecific inflammatory reaction. In contrast to GCL, cells in BEC do not have Birbeck granules, do not express CD1a; they have positive immunohistochemical reaction with antibodies to CD68, CD163 and clotting factor XIIIa, and some of them to S 100 [12,13,14]. The final diagnosis is usually based on three components: clinical symptoms, biopsy results and radionuclide skeletal scanning.

The tissue tropism of BEC is not fully understood, but many of the lesion sites are rich in adipose tissue, such as bone marrow and the paranephral region [15]. BEC involves the heart in 40–75% of cases [16] and can manifest with typical involvement of bone, the central nervous system, retro-orbital tissue, lung, liver, spleen, retroperitoneal tissue and skin. Clinical manifestations include non-sugar diabetes if the pituitary gland is affected, dyspnoea secondary to lung or heart damage, exophthalmos, ataxia, rash, fatigue, weight loss and bone pain.

The xanthogranulomas (CG) family is diseases with various clinical manifestations ranging from solitary to multiple or disseminated with lesions in different areas of the body and different patient ages [17]. CG is the most common of the non-Langerhans cell histiocytoses. It is a benign disease in which one, a few or sometimes multiple red or yellow nodules are present, ranging from 0.5 to 1.0 cm in diameter, which usually resolve spontaneously within months or years. Most lesions appear in the first few years of life. Extracutaneous and disseminated juvenile xanthogranulomatosis (JXG) are discussed in group L. Adult xanthogranuloma (AGX) is usually solitary and persistent. Solitary reticulohistiocytoma is a xanthogranuloma dominated by oncocytic macrophages and giant ‘frosted glass’ cells. Histologically, the above xanthogranulomas appear as well-defined dermal or dermo-hypodermal nodules that do not involve the epidermis. Mature foci contain foam cells, giant foreign body cells, and Tuton cells, as well as macrophages, lymphocytes, and eosinophils. Older, regressing lesions show fibroblast proliferation and fibrosis, which replace part of the infiltrate.

Monocytes and macrophages, as the most important cells of the innate immune system, play an essential role in the body’s defence against viral infections. They mainly respond to microbial antigens by producing inflammatory mediators to remove pathogens and repair tissue damage. However, aberrant changes in their function, such as cytokine storm, can be very detrimental to the host in cases of SARS-CoV-2-induced acute respiratory distress syndrome. Moreover, the inflammatory responses stimulated by SARS-CoV-2 have also affected other vital organs of the body, including the heart [18]. On the other hand, mild COVID-19 leads to impaired effector functions of macrophages and eicosanoid metabolism, causing long-term immune disturbances in convalescent patients [19]. Finally, the possibility of SARS-CoV-2 to persist in macrophages along with endothelium, cardiomyocytes, and fibroblasts has been discussed [20]. The objective of the study: To demonstrate extremely rare cases of CD68-negative cardiac histiocytosis with expression of SARS-CoV-2 antigen in infiltrate cells.

## 2. Results

### 2.1. Clinical Case 1

#### 2.1.1. Clinical Data

**Figure 1:**
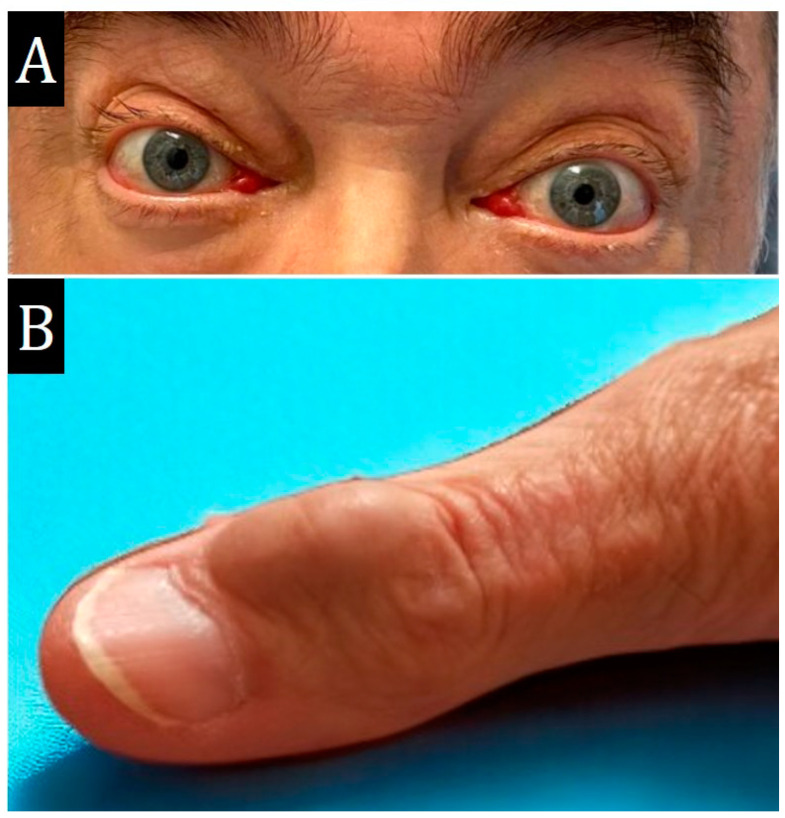
External examination of the patient on admission. (**A**)—pronounced exophthalmos. (**B**)—thumb mass.

A 67-year-old man with exophthalmos, exudative pericarditis and a dense nodular skin mass on the middle phalanx of the 1st finger of the hand, 2 × 1.5 cm (Figure 1), had a 30-year history of joint syndrome with predominant involvement of the sacroiliac joint and hip joint. Observed by a rheumatologist, the main markers were negative. In February 2021 and in July 2022, he had a mild coronavirus infection.

#### 2.1.2. Radiation Diagnostic and Echocardiographic Data

**Figure 2:**
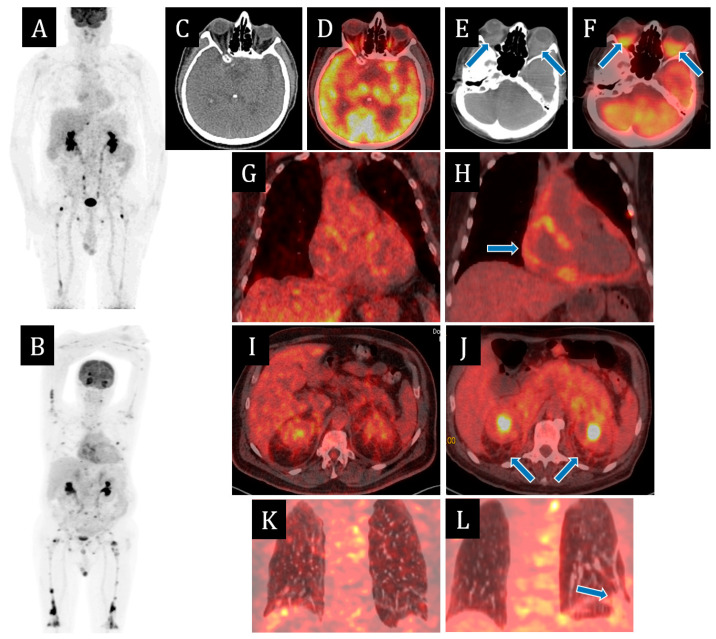
Positron emission tomography/computed tomography (PET/CT) images with [18F]-fluorodeoxyglucose ([18F]-FDG) on 9 March 2022 (**A**,**C**,**D**,**G**,**I**,**K**) and on 27 July 2023 (**B**,**E**,**F**,**H**,**J**,**L**). The maximum intensity projection (MIP) images (**A**,**B**) summarize the physiological and pathological distribution of ([18F]-FDG) in the body. PET/CT scans on 27 July 2023 showed the negative dynamics in the form of metabolically active changes in retrobulbar fibres (**E**,**F**), appearance of metabolic active pericardial effusion (**H**) and consolidation zones in S9, S10 of the left lung (**L**), the increase in the number and metabolic activity of foci in skeletal bones (**B**) and the increase of pronounced changes in retroperitoneal fibre (**J**).

Since 2021, multispiral computed tomography (MSCT) of the chest organs for the first time revealed minimal effusion in the pericardial cavity. According to echocardiography from March 2022, there was divergence of pericardial sheets of up to 5–7 mm; a year later, of up to 16 mm behind the lower-lateral and lateral walls of the left ventricle (LV) and up to 14 mm behind the right heart chambers; also, fibrin deposits of up to 11 mm thick. The contractile function of the LV and right ventricle (RV) were preserved. MSCT of abdominal cavity organs with contrast dated March 2022 revealed thickening of paranephral and retroperitoneal fibres. Positron emission tomography (PET CT) of the whole body with 18F-fluorodeoxyglucose in the same month revealed signs of small focal lesions of skeletal bones with a lithic replacement component on CT, with hyperfixation of radiopharmaceutical (RFP; Figure 2). On scintigraphy two months later, there were signs of diffusely increased RFP accumulation in the projections of large and small joints of upper and lower extremities, diffusely increased RFP accumulation in the projections of diaphyses of femoral bones in the lower third and tibia bones throughout. Myelogram was performed—no pathology was detected.

Oncomarkers AFP CA 19-9 CEA, PSA from March 2022 were within the reference values. Progression of heart failure (HF) clinic to functional class IV (FC) was observed in the dynamics. An echocardiographic study two months later revealed a left ventricular ejection fraction (LVEF) of 81% (Simpson), fibrin deposits on the visceral pericardial leaflet, divergence of the leaflets behind the LV and LV from 15 to 44 mm with signs of pretamponade, in connection with which pericardiocentesis, fenestration and pericardial biopsy were performed in St. Petersburg hospital two months later; a histological examination revealed a pericardial fragment with productive and fibrinous inflammation.

In the same month, the patient was admitted to the cardiology department of the Almazov NMMC of the Russian Ministry of Health with the clinical diagnosis of severe biventricular HF and conduction disturbances in the form of atrioventricular block II degree 2 type, with episodes of atrioventricular substitution rhythm. Cardiac MRI showed no evidence of accumulation disease, myocarditis, or cardiac sarcoidosis.

#### 2.1.3. Morphological Examination Data

On reviewing the pericardial biopsy data, histological examination showed that the pericardium was thickened due to infiltration by large cells with a wide rim of eosinophilic cytoplasm, with giant multinucleated cells, with a small number of plasma cells, lymphocytes and leukocytes.

**Figure 3:**
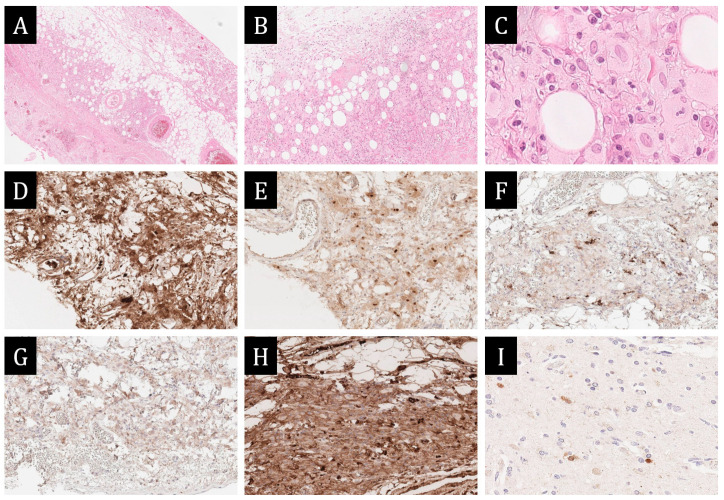
Morphological study of the pericardium. (**A**,**B**)—infiltration of pericardial adipose tissue by xanthoma cells, ×40, ×100. (**C**)—histiocytic phenotype of the infiltrate: large polygonal cells with pale eosinophilic cytoplasm and oval light-coloured nucleus and irregular contours of nuclear membranes, ×800. (**D**–**H**)—immunohistochemical study ×200: (**D**)—diffuse staining of infiltrate cells with S100; (**E**)—nuclear expression of cyclinD1; (**F**)—expression of CD38 on single infiltrate cells; (**G**)—weak expression of CD68 on single cells; (**H**)—intense expression of CD163 on most of the infiltrate cells. (**I**)—focal moderate granular cytoplasmic expression of spike protein SARS-CoV-2 in infiltrate cells, ×400.

Immunohistochemical examination (IHC) revealed that large cells did not express langerin, mesotelin, ALK and CK20. There were 6 CD38+/IgG+ plasmacytes in 1 HPF p/view that had concomitant expression of IgG4 (IgG = IgG4). Large cells expressed cyclinD1, CD163 and S100, with single cells weakly expressing CD68. Background lymphocytic infiltration was represented by CD20+ lymphocytes; in a smaller part, nuclear expression of Ki-67 was determined. It is also worth noting that many of the large cells of the infiltrate, including single giant cells, had focal moderate granular cytoplasmic expression of spike protein SARS-CoV-2 (Figure 3). Based on the histological picture and immunophenotype, a diagnosis of xanthogranulomatous pericarditis was made. The morphological picture was not inconsistent with Erdheim–Chester disease. Langerhans histiocytosis, sarcoidosis, and IgG4-associated disease were excluded. Genetic testing of histological material paraffin blocks for BRAF, NRAS, PIK3CA mutations was negative.

According to PET CT with 18F-fluorodeoxyglucose, negative dynamics was observed in the form of metabolically active changes in retrobulbar, posterior mediastinal, presacral and parapancreatic fibres, increase of pronounced changes in retroperitoneal and paraneural fibre, increase in the number and metabolic activity of foci in skeletal bones, appearance of metabolically active consolidation zones in S9, S10 of the left lung and interstitial changes in supradiaphragmatic sections of S9, S10 of the right lung (Figure 2B).

**Figure 4:**
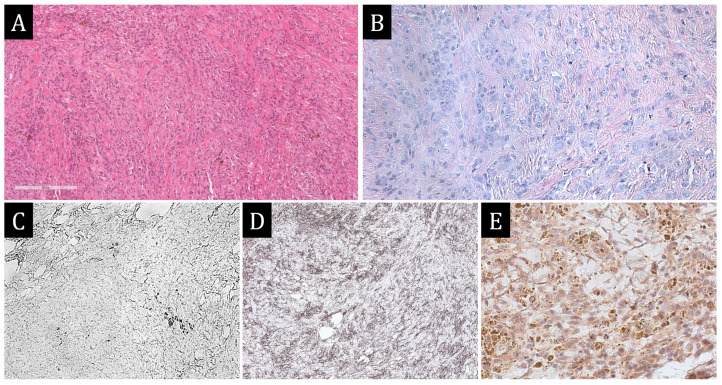
Morphological study of the thumb mass. (**A**,**B**)—monomorphic infiltrate with predominance of cells of histiocytic morphology on the background of collagen stroma: (**A**)—×100, (**B**)—×200. (**C**,**D**)—immunohistochemical study, ×100: (**C**)—absence of CD68 expression by infiltrate cells; (**D**)—intense membrane expression of CD163. (**E**)—granular cytoplasmic expression of spike protein SARS-CoV-2 in infiltrate cells, ×400.

A biopsy of the finger mass was performed for histological verification. Morphological examination under the preserved epidermis in the dermis revealed infiltration of large cells with a wide rim of cytoplasm similar to that in the pericardium. The immunophenotype of the cells matched the infiltrate in the pericardium. All of them expressed CD163, and many of them expressed the spike protein SARS-CoV-2 in the cytoplasm (Figure 4).

Taking into account the progression of clinical symptoms—the clinical diagnosis of chronic heart failure, significant effusion in the pericardial cavity, cardiac conduction disorders, increased exophthalmos—glucocorticosteroids and tumour necrosis factor alpha inhibitor were prescribed. Pulse therapy with GCS (methylprednisolone) was carried out, with a switch to tablet forms of prednisolone with recommendations for reduction in dynamics, and infusions of infliximab (400 mg) were performed three times. Before the results of the genetic study were obtained, pulse therapy with GCS (methylprednisolone) was performed in order to control the inflammatory process, with a switch to tablet forms of prednisolone with recommendations for reduction in dynamics; infusions of infliximab (400 mg) were performed three times.

After receiving the results of the genetic study, therapy with vemurafenib was initiated from a starting dose of 240 mg twice daily. Taking into account good tolerability, absence of side effects, and severity of the clinical picture, the dose was escalated to 480 mg three times a day (1440 mg) together with continuation of therapy with prednisolone with a positive clinical and laboratory effect.

In addition, therapy of heart failure was commenced in the form of ACE inhibitors. The dynamics, conduction disturbances regressed, but pericardial effusion and the clinical picture of HF at the level of III class, and exophthalmos, progressed.

#### 2.1.4. Genetic Testing

A DNA sample from thumb granuloma cells (native material) revealed a nucleotide sequence variant in the BRAF gene c.1799T>A (p.Val600Glu) by direct Sanger sequencing of the BRAF gene coding sequence (exon 15), transcript NM 004333.6. A definitive diagnosis of Erdheim–Chester disease was made. After that, the therapy with vemurafenib, a BRAF-kinase inhibitor, was started.

#### 2.1.5. Treatment Regimen

Therapy with vemurafenib was initiated with a starting dose of 240 mg twice daily. Taking into account good tolerability, absence of side effects, and severity of the clinical picture, the dose was escalated to 480 mg three times a day (1440 mg) together with the continuation of therapy with prednisolone with positive clinical and laboratory effect.

As part of the prevention of infectious complications against the background of combined immunosuppressive therapy, sulfomethoxazole + trimethoprim, also acyclovir therapy was carried out.

In addition, therapy of heart failure in the form of ACE inhibitors (perindopril), loop diuretics (torasemide), mineralocorticoid receptor antagonists (eplerenone) was constantly performed. Therapy with SGLT-2 inhibitors was not performed due to a history of urinary tract infection.

In 4 months, a significant positive response to the therapy was obtained in the form of regression of exophthalmos and effusion in the pericardial cavity—fibrinous content up to 9 mm, compensation of the clinical picture of CHS at the level of II FC, according to PET CT—reduction of size and metabolic activity of retrobulbar fibres of both orbits, mediastinal, abdominal cavity and retroperitoneum, retroperitoneal, paranephral and presacral fibres, reduction of metabolic activity of pericardial sheets, and bone and lung foci (Figure 2). The patient continued the therapy with BRAF-kinase inhibitor and glucocorticosteroids.

### 2.2. Clinical Case 2

A 63-year-old man was admitted to hospital with progressive chronic heart failure due to constrictive pericarditis with cardiomegaly and complex cardiac rhythm disturbances. The last suffered COVID-19 seven months prior to the current hospitalisation.

#### Morphological Examination Data

**Figure 5:**
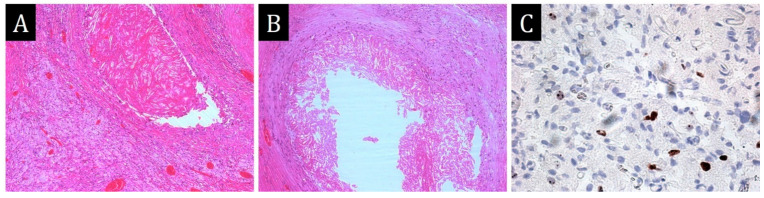
Morphological study of pericardium. (**A**,**B**)—non-necrotizing xanthogranulomatous pericardial inflammation with large deposits of cholesterol crystals in central sections, ×100. (**C**)—granular expression of spike protein SARS-CoV-2 in the cytoplasm part of granuloma cells, ×200.

Fenestration with a pericardial biopsy was performed, which revealed multiple fibrin overlays and foci of granulomatous inflammation with cholesterol crystal deposits in the centre surrounded by macrophages and haemosiderophages. All cells expressed CD163, and some of them expressed the spike protein SARS-CoV-2. A diagnosis of xanthogranulomatous pericardium was made (Figure 5).

In 1 week, the patient died of ventricular fibrillation. The autopsy showed signs of decompensation of congestive heart failure: left hydrothorax—350 mL, ascites—300 mL, alveolar pulmonary oedema, acute and chronic venous haemorrhage of the liver, kidneys, and spleen. Symmetric myocardial hypertrophy was detected (heart mass—520 g, left ventricular wall thickness—1.8 cm, LVH thickness—1.8 cm). No thromboembolic complications were identified.

**Figure 6:**
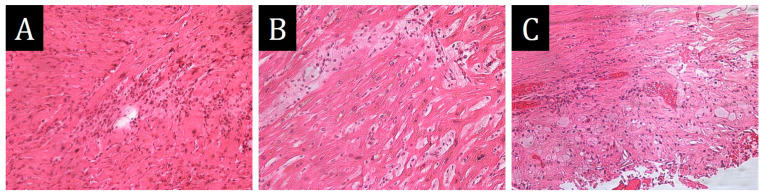
Morphological study of autopsy material of myocardium and pericardium, ×200. (**A**)—aggressive polymorphocellular infiltrate in myocardial interstitium with muscle fibre infiltration and necrosis. (**B**)—perimuscular fibrosis, muscle fibre discomplementation, single perinuclear vacuoles in sarcoplasm of cardiomyocytes. (**C**)—infiltration of pericardium by frothy histiocytes with admixture of lymphocytes and plasmocytes.

The histological study revealed dyscomplexation of muscle fibres, hypertrophy and lipofuscinosis of cardiomyocytes, marked polymorphism and hyperchomatosis of cardiomyocyte nuclei, perinuclear vacuoles. The walls of intramural arteries were sharply thickened due to fibrosis. The expressed diffuse polymorphocellular stromal infiltration was represented by lymphocytes, histiocytes, neutrophils, with point necrosis of cardiomyocytes. Small focal perimuscular fibrosis was observed (Figure 6). The final diagnosis was hypertrophic cardiomyopathy with polymorphonuclear active myocarditis and pericardial xanthogranulomatosis; the complication was progressive chronic heart failure. No xanthogranulomatous reaction was detected in other organs. The genetic study was not performed.

### 2.3. Clinical Case 3

A 1-month-old girl, 51 cm tall, weighing 3.4 kg was admitted to the hospital with a diagnosis of unspecified cardiomyopathy, acute myocarditis, terminal chronic heart failure with complex heart rhythm disturbances for follow-up examination and further treatment. From the anamnesis, it is known that the child is from the first pregnancy of a 26-year-old mother without diagnosed cardiovascular pathology with an unexploited hereditary history of cardiovascular disease. The course of pregnancy was complicated against the background of acute respiratory viral infection and low water supply. Cervicitis associated with ureaplasma was diagnosed in the mother during gestation. In the anamnesis, 2 years before the pregnancy there was a mild course of COVID-19. The child died one day after hospitalization from ventricular fibrillation, while the mother’s postpartum period was without complications.

#### Morphological Examination Data

At autopsy, the pericardial cavity contained clear yellow fluid in the volume of 160 mL. The heart measured 6.5 × 5.0 × 4.2 cm, weighing 95 g. Myocardium of flabby consistency. There was a small amount of blood clots in the heart cavities. The heart cavities were not dilated. Left ventricular wall thickness in the middle third was 0.6 cm, right ventricular wall thickness was 0.3 cm, and interventricular septum thickness was 0.7 cm. Myocardial hypertrabecularity was determined in all sections of all left ventricular walls: true myocardial thickness—0.6 cm, trabeculae thickness—1.4 cm. On the section of myocardium of papillary muscles, left ventricular walls with multiple irregularly shaped foci of grey colour up to 2.0 × 1.5 cm in size. Myocardium of interventricular septum was subtotally grey. The endocardium was smooth, thin transparent, and shiny. The valve leaflets were unchanged. Atrial cavities were not dilated. Coronary arteries receded on the section. The perimeter of the right coronary artery mouth was 0.7 cm, and that of the left coronary artery was 0.8 cm.

**Figure 7:**
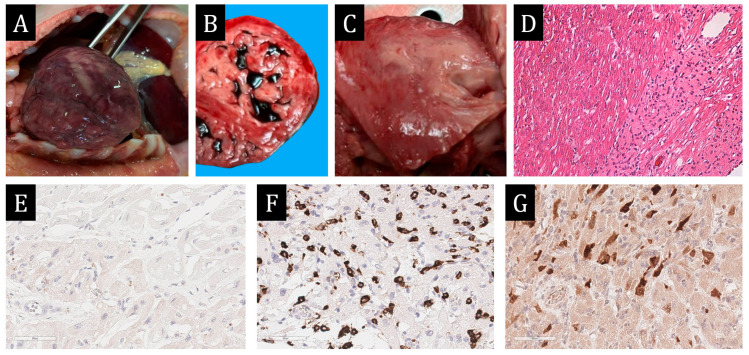
Autopsy study. (**A**)—marked cardiomegaly. (**B**)—hypertrabecularity of the left ventricular myocardium (the ratio of noncompact layer to compact layer exceeds 2:1). (**C**)—myocardium of the interventricular septum on the section: irregularly shaped yellowish-grey areas of tissue bulging above the cut surface are visualised. (**D**)—fields of polygonal light eosinophilic cells with nuclear morphology of histiocytes among the working myocardium, H&E × 100. (**E**)—absence of CD68 staining in the fields of histiocytic cells. (**F**)—intense expression of CD163 in most of the histiocytic cells. (**G**)—expression of spike protein SARS-CoV-2 in some of the histiocytic cells. (**E**–**G**)—×400.

Histological and IHC of the myocardium revealed multiple mosaic hemorrhagic necroses and zones of aggressive lymphocytic infiltration with more than 14 CD3+ cells per 1 mm^2^. Also in the trabecular muscles, in the thickness of the left ventricle and interventricular septum, large polygonal cells with broad eosinophilic, frothy cytoplasm were detected. The cells were arranged in fields, sometimes chaotically (Figure 7).

The above morphological picture necessitated differential diagnosis with multiple myocardial rhabdomyomas, myocardial xanthogranulomatosis and histiocytoid cardiomyopathy. In favour of histiocytoid cardiomyopathy in this patient were evidence of death via ventricular arrhythmias, similar localisation and macroscopic pattern of myocardial lesions. Rhabdomyoma was favoured by multifocal lesions, thickening of the noncompact layer of myocardium, similar histological picture: abundance of large polygonal cells with light cytoplasm. However, it should be noted that the so-called ‘spider cells’ characteristic for this tumour were not found in the myocardium. Myocardial xanthogranulomatosis was favoured by multifocal lesions, pericardial effusion, and the development of ventricular rhythm disturbances.

IHC was performed for differential diagnosis, and the results revealed the immunophenotype of the cells to be S100+, CD163+, spike protein SARS-CoV-2‘+’, myosin‘−’, desmin‘−’, connexin 43‘−’, CD45‘−’. Single cells were stained with CD68. In addition, tubular hypoplasia of the descending thoracic aorta (perimeter of the ascending aorta—3.6 cm, descending aorta—2.4 cm, abdominal aorta—3.5 cm), open oval window with a diameter of 0.6 cm, signs of acute and chronic venous hemorrhage of the liver, kidneys, spleen, ascites (400 mL), hydrothorax (500 mL on the right, 600 mL on the left), secondary pulmonary hypertension. No thromboembolic complications were identified. The final diagnosis was: Noncompact left ventricular myocardium, hypoplasia of the descending thoracic aorta, active lymphocytic myocarditis, and myocardial xanthogranulomatosis. Complication: Progressive chronic heart failure, secondary pulmonary hypertension. The genetic study was not performed.

### 2.4. Clinical Case 4

A 57-year-old man was admitted to the hospital with the clinical picture of constrictive pericarditis, stage III hypertension and progressive heart failure. At echocardiographic examination, the pericardium was thickened, and the pericardial sheets were fused behind the posterolateral portions of the left ventricle. There was marked dilatation of the right heart chambers and left atrium. The total contractility of the left ventricular myocardium was reduced due to diffuse hypo- and akinesia (LV EF according to Simpson 42%). Sternotomy and pericardectomy were performed over the left and right ventricles, their diaphragmatic surfaces and the lateral wall of the left ventricle. On examination of the operative material, the pericardium was thickened up to 1 cm, with fibrinous deposits and focal calcinosis.

#### Morphological Examination Data

**Figure 8:**
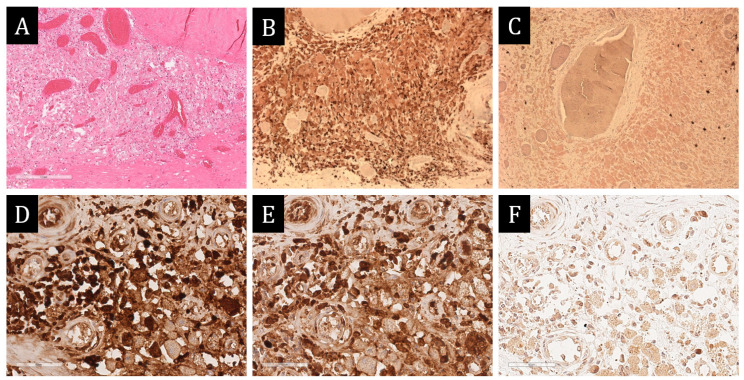
Morphological study of pericardium. (**A**–**C**)—×100, (**D**–**F**)—×400. (**A**)—histiocytic infiltrates of pericardium. (**B**)—expression of CD163 in all infiltrate cells. (**C**)—background CD38 + plasmocytes. (**D**)—expression of IgG in part of infiltrate cells. (**E**)—expression of IgG4 in the majority of IgG + plasmocytes. (**F**)—expression of spike protein SARS-CoV-2 in the majority of infiltrate cells.

Histological and immunohistochemical examination revealed large-cell infiltrates with and without obvious fat content in association with plasmocytes among fibrous tissue. Diffuse bright CD163 expression was detected in large cells and CD38 expression in single cells, CD38 expression in plasmocytes, and IgG4 expression in more than 10 plasmocytes in 1 HPF (IgG4 expression in 192 plasmocytes per field of view, IgG expression in 240 plasmocytes per field of view). Single cells were stained with CD68. Expression of the spike protein SARS-CoV-2 was detected in the majority of macrophages (Figure 8). Pathologists made a diagnosis of chronic xanthogranulomatous pericarditis. The results of histological and immunohistochemical examination made it possible to exclude tuberculosis, sarcoidosis, and IgG4-associated disease.

Further skeletal bone scintigraphy revealed arthrosis of the right acromioclavicular joint, metatarsophalangeal joints of the big toes, and symmetrical increase of RFP accumulation in the distal metaphyses of the femurs, proximal and distal metaphyses of the tibia. No infiltrative changes were detected on chest radiography in 2 projections. Moderate pneumofibrosis, signs of pulmonary hypertension, and S10 hypoventilation on the left were noted. The pulmonary artery was dilated. With clinical improvement (regression of chronic heart failure clinic), the patient was discharged for outpatient treatment, and a paraffin block of pericardium was sent for the genetic study of mutation in the BRAF gene in order to exclude Erdheim–Chester disease.

## 3. Discussion

In all four cases, we found granulomatous inflammation of the heart. Three of them had pericardial involvement, and two had myocardial involvement. In one case, a diagnosis of Erdheim–Chester disease was made. This systemic histiocytosis is thought to be characterised by xanthomatous infiltration of tissues by lipid-laden histiocytes [21,22]. As in other xanthogranulomatous diseases, rare giant Touton cells (giant cells containing foamy cytoplasm) are present in the infiltrate, and the lipid-laden histiocytes in the lesion focus are CD1‘−’ and CD68+. However, in our case, only single histiocytes expressed CD68, and all giant multinucleated cells were CD68‘−’, while all of them expressed CD163. The granulomas in the other three cases had exactly the same immunophenotype.

CD68 is a highly glycosylated type-I transmembrane glycoprotein that is mainly bound to the endosomal/lysosomal compartment but can rapidly translocate to the cell surface [23,24]. The role of CD68 in inflammation and immunity is still a mystery, and its role as a scavenger receptor remains to be confirmed. CD68 is not thought to be involved in bacterial/viral pathogen binding, innate, inflammatory or humoral immune responses, although it could potentially be involved in antigen processing/presentation, while macrophages devoid of CD68 show normal phagocytic activity and do not exhibit a defective innate response to infection; they have a normal ability to present antigens and induce a humoral immune response [25].

M2 CD163 + macrophages are known to be involved in the profibrotic state observed in the context of fibrosis in many tissues [26,27]. In all our cases, CD163 + macrophages expressed the spike protein SARS-CoV-2. Bost P. et al. [28] also detected SARS-CoV-2 transcripts in macrophages from patients with severe COVID-19 infection. Negative-strand mRNA readouts indicating some level of viral replication allowed Grant RA et al. to detect SARS-CoV-2 in some subclusters of macrophages, including tissue-resident macrophages [29]. Whether infected macrophages produce newly synthesised viral particles (so-called productive or abortive infection) is a matter of debate and may be related to the origin of macrophages. In any case, the apparent profibrotic polarising effect of direct infection of monocytes with SARS-CoV-2 opens the way for the investigation of potentially suitable drugs [30].

Thus, the absence of CD68 expression in the majority of granulomatous inflammatory cells in our cases can be explained by viral load in the conditions of the COVID-19 pandemic [31]. In our opinion, this fact confirms virus persistence in macrophages, which is consistent with the opinion of other authors [32]. Therefore, it is recommended to perform immunohistochemical study with antibodies to CD163 and SARS-CoV-2 in cases of granulomatous myopericarditis detection.

Cytokine storm is characterized by rapid proliferation and increased activity of T cells, macrophages, and natural killer cells with the release of various inflammatory cytokines and chemical mediators by protective cells [33,34]. Histiocytoses are also characterized by the accumulation of activated macrophages, dendritic cells or cells of monocytic origin in various tissues and organs. Thus, the synergy of COVID-19 infection and histiocytosis is appropriate, and SARS-CoV-2 may be a trigger of disease progression, including Erdheim–Chester disease, which occurred in our relapsed patient who had had this infection twice.

In addition, examining biopsy, surgical and autopsy material during the last 30 years of work in a specialised cardiology centre, it was in the post-COVID period that we first encountered granulomatous lesions of the heart. All four cases described were identified in the last year. While the first case is clinically, genetically, and histologically confirmed as Erdheim–Chester disease, the others represent localized granulomatosis represented mainly by M2 macrophages with expression of the spike protein SARS-CoV-2, which in our opinion can be interpreted as an inflammatory response to viral infection.

The clinical picture and instrumental examination data fully correspond to the description of similar cases of Erdheim–Chester disease in the literature [35]. Vemurafenib was approved by the U.S. Food and Drug administration for treatment of BRAF-mutated ECD [36]. Our case confirms the successful treatment of the disease with this drug. In particular, we observed regression of exophthalmos, CHF clinical picture, and pericardial effusion, but the treatment with this drug is continued, as we have received only partial response to therapy so far.

Cardiac xanthogranulomas are also extremely rare and are mainly found in children [37]. Moreover, they are more often found in the pericardium rather than in the myocardium. Therefore, we had to differentiate myocardial granulomatosis in a 1-month-old patient with rhabdomyoma and histiocyte-like cardiomyopathy (Purkinje cell hamartoma).

In addition, we found only one case report in the literature of a CD68-negative non-lipidised juvenile xanthogranuloma in a 7-year-old boy, but of a different localisation (on the forehead) [38]. To our knowledge, juvenile cardiac xanthogranulomas in adults have been described only twice in the literature [39,40], and before the COVID-19 pandemic.

Our experience shows that, in any case of histiocytosis detection, genetic testing should be performed for subsequent targeted therapy, as was the case with the first patient. It is better to test native material rather than paraffin blocks of tissue. Our two deceased patients did not have time to undergo genetic analysis because of their short hospital stays.

## 4. Materials and Methods

The material for morphologic study was collected by pericardial fenestration (anterior wall), EMB (typical sites of myocardium of the right ventricle and interventricular septum), or autopsy (fragments of myocardium of the lateral walls of the right and left ventricles were presented in the study). The routine histological examination of the myocardium was carried out with hematoxylin and eosin staining in all myocardial samples.

IHC was carried out according to a standard protocol using antibodies against the following targets: CD68 (mouse monoclonal antibody, clone PG-M1; DAKO, Carpinteria, CA, USA; dilution 1:100); spike protein SARS-CoV-2 (rabbit polyclonal antibody, GeneTex, Hsinchu City, Taiwan, dilution 1:50); CD38 (rabbit monoclonal antibody, clone SP149, CELL MARQUE, Belgium, The Netherlands, dilution 1:100); IgG (rabbit polyclonal antibody, DAKO, Glostrup, Hovedstaden, Denmark, dilution 1:10,000); IgG4 (rabbit monoclonal antibody, clone EP138, EPITOMICS, Burlingame, CA, USA, dilution 1:50); CD163 (rabbit monoclonal antibody, clone EPR19518, Abcam, Cambridge, MA, USA, dilution 1:100); Cyclin D1 (rabbit monoclonal antibody, clone SP4, Diagnostic BioSystems, Pleasanton, CA, USA, dilution 1:50); S100 (rabbit polyclonal antibody, DAKO, Glostrup, Hovedstaden Denmark, dilution 1:600).

Morphometric analysis was carried out on scanned histology slides using the Aperio ImageScope v12.3.3 software.

## 5. Conclusions

In this article, we demonstrated four cases of extremely rare CD68-negative cardiac xanthogranulomatosis in three adults and one child with expression of the spike protein SARS-CoV-2 in M2 macrophages: A case of Erdheim–Chester disease in a 67-year-old man with pericardial involvement and positive dynamics with vemurafenib treatment, an autopsy case of xanthogranulomatous myopericarditis in a 63-year-old man, surgical material of xanthogranulomatous constrictive pericarditis in a 57-year-old man, and an autopsy case of xanthogranulomatosis in a 1-month-old girl. The diagnosis of histiocytosis involving the myocardium presents a significant challenge for pathologists. Accurate diagnosis necessitates a thorough assessment of systemic involvement. Critical to this process is an extensive differential diagnosis that distinguishes histiocytosis from other conditions such as autoimmune myocarditis and pericarditis, IgG4-related diseases, sarcoidosis, tuberculosis, Wegener’s granulomatosis, and other granulomatous conditions affecting myocardial tissue. Histiocytosis should be considered a diagnosis of exclusion, requiring comprehensive morphological evaluation and immunohistochemical analysis. Essential markers for this analysis include CD68, CD1a, langerin, CD45, S100, CD163, CD80, CD20, CD38, clotting factor XIIIa, IgG, and IgG4. Given the complexity and the need for a multidisciplinary approach, accurate diagnosis of histiocytosis not only requires advanced pathological techniques, genetic testing but also a thorough understanding of the patient’s clinical history and systemic presentation.

This potential indirect association between COVID-19 and the development of histiocytosis in these patients warrants further investigation. To confirm this hypothesis, a large sample of patients is needed, involving all morphologic techniques and mandatory genetic testing.

## Data Availability

Availability of data and materials. All source data are are included in the article. If you need clarifications, or need additional information, you can write to the email: doctormakarovia@gmail.com.
